# Correlation of multiple lipid and lipoprotein ratios with nonalcoholic fatty liver disease in patients with newly diagnosed type 2 diabetic mellitus: A retrospective study

**DOI:** 10.3389/fendo.2023.1127134

**Published:** 2023-02-17

**Authors:** Ran Li, Dehong Kong, Zhengqin Ye, Guannan Zong, Kerong Hu, Wei Xu, Ping Fang, Liya Zhang, Yun Zhou, Keqin Zhang, Ying Xue

**Affiliations:** Department of Endocrinology and Metabolism, Tongji Hospital, School of Medicine, Tongji University, Shanghai, China

**Keywords:** lipid ratios, lipoprotein ratios, TG/HDL-C ratio, NAFLD, newly diagnosed T2DM

## Abstract

**Background and objective:**

The diagnostic value of lipid and lipoprotein ratios for NAFLD in newly diagnosed T2DM remains unclear. This study aimed to investigate the relationships between lipid and lipoprotein ratios and the risk of NAFLD in subjects with newly diagnosed T2DM.

**Methods:**

A total of 371 newly diagnosed T2DM patients with NAFLD and 360 newly diagnosed T2DM without NAFLD were enrolled in the study. Demographics variables, clinical history and serum biochemical indicators of the subjects were collected. Six lipid and lipoprotein ratios, including triglycerides to high-density lipoprotein-cholesterol (TG/HDL-C) ratio, cholesterol to HDL-C (TC/HDL-C) ratio, free fatty acid to HDL-C (FFA/HDL-C) ratio, uric acid to HDL-C (UA/HDL-C) ratio, low-density lipoprotein-cholesterol to HDL-C (LDL-C/HDL-C) ratio, apolipoprotein B to apolipoprotein A1 (APOB/A1) ratio, were calculated. We compared the differences in lipid and lipoprotein ratios between NAFLD group and non-NAFLD group, and further analyzed the correlation and diagnostic value of these ratios with the risk of NAFLD in the newly diagnosed T2DM patients.

**Results:**

The proportion of NAFLD in patients with newly diagnosed T2DM increased progressively over the range Q1 to Q4 of six lipid ratios, including the TG/HDL-C ratio, TC/HDL-C ratio, FFA/HDL-C ratio, UA/HDL-C ratio, LDL-C/HDL-C ratio, and APOB/A1 ratio. After adjusting for multiple confounders, TG/HDL-C, TC/HDL-C, UA/HDL-C, LDL-C/HDL-C and APOB/A1 were all strongly correlated with the risk of NAFLD in patients with newly diagnosed T2DM. In patients with newly-onset T2DM, the TG/HDL-C ratio was the most powerful indicator for the diagnosis of NAFLD among all six indicators, with an area under the curve (AUC) of 0.732 (95% CI 0.696–0.769). In addition, TG/HDL-C ratio>1.405, with a sensitivity of 73.8% and specificity of 60.1%, had a good diagnostic ability for NAFLD in patients with newly diagnosed T2DM.

**Conclusions:**

The TG/HDL-C ratio may be an effective marker to help identify the risk of NAFLD in patients with newly diagnosed T2DM.

## Introduction

Non-alcoholic fatty liver disease (NAFLD) and type 2 diabetes mellitus (T2DM) have a strong bidirectional association, and the prevalence of both is increasing simultaneously (1, 2). A recent meta-analysis reported the global prevalence of NAFLD in patients with T2DM was 55.5% (2). Moreover, the global prevalence of T2DM in patients with NAFLD and non-alcoholic steatohepatitis (NASH) patients was 22.51%, and 43.63%, respectively (3). There is now growing evidence that patients with T2DM combined with NAFLD tend to have poorer glycemic control than T2DM patients without NAFLD, and are at higher risk of developing NASH, cirrhosis or even hepatocellular carcinoma compared to NAFLD patients without T2DM (4). On the other hand, the incidence of chronic diabetic complications, such as cardiovascular disease (CVD), chronic kidney disease (CKD) and retinopathy, is also significantly higher in patients with T2DM combined with NAFLD than in those without combined NAFLD (4, 5).

Liver biopsy is the gold standard for the diagnosis of NAFLD and NASH cirrhosis. However, in clinical practice, the invasiveness, poor acceptability and high cost of liver biopsy make it difficult to use for widespread screening in the general population (6, 7). Conventional ultrasonography is commonly used for screening and diagnosis of NAFLD (7). However, due to the large number of patients with T2DM, routine liver ultrasound screening in all T2DM patients requires extremely expensive medical expenses. In addition, a large number of rural health centers or community hospitals lack ultrasound equipment and qualified ultrasonographers. Therefore, several previous studies have pinned hopes for early screening of patients with NAFLD on various serum markers (8–13). However, to date, no serum marker has become an accepted diagnostic indicator for NAFLD.

It is well known that serum biochemical indices of routine physical examination include liver enzymes and blood lipids. Previous studies have shown that liver enzyme levels are not useful for screening for NAFLD as their changes do not necessarily correspond to the degree of hepatic steatosis (14). Dyslipidemia, including increases in triglycerides (TG), cholesterol (TC), low-density lipoprotein-cholesterol (LDL-C) and decreases in high-density lipoprotein-cholesterol (HDL-C), is strongly associated with NAFLD (11, 13, 15, 16). Several current data have indicated that lipid and lipoprotein ratios are more valuable than individual lipid values in predicting the risk of T2DM or NAFLD because they can reflect the interaction between lipid components (11–13, 15–17). Among them, the ratios of TG to HDL-C (TG/HDL-C) (12, 13), TC to HDL-C (TC/HDL-C) (11), uric acid (UA) to HDL (UA/HDL-C) (9), LDL-C to HDL-C (LDL-C/HDL-C) (16) and apolipoprotein B to apolipoprotein A1 (APOB/A1) (17) have been previously reported to be associated with the risk of NAFLD in different populations. Besides, TG/HDL-C and TC/HDL-C have been described as promising parameters for the diagnosis of prediabetes and T2DM (15, 18).

Currently, there are no studies on the relationship between the aforementioned lipid and lipoprotein ratios and NAFLD in a newly diagnosed T2DM population with no history of medication and no diabetic complications. Considering the high prevalence and risk of combined NAFLD in T2DM, there is a need for early identification of NAFLD in newly diagnosed diabetic patients for better early intervention. Therefore, this study sought to evaluate the value of the above-mentioned lipid-lipoprotein ratios for assessing the risk of NAFLD in patients with newly diagnosed T2DM.

## Methods

### Participants

This study was a retrospective study approved by the Ethics Committee of Tongji Hospital, Tongji University School of Medicine (K-2021-010). A total of 1021 patients who were first diagnosed with T2DM and not receiving anti-diabetic medication at the inpatient department of the Department of Endocrinology, Tongji Hospital, Tongji University, from June 2018 to December 2020 were enrolled.

The diagnosis of T2DM was based on the criteria of the World Health Organization (1999) (19). The diagnosis of NAFLD was made by abdominal ultrasound assessment of hepatic steatosis (20). The criteria were as follows: 1) diffusely enhanced liver echogenicity that was stronger than that of the kidneys or spleen; 2) attenuation of far-field echogenicity depth in the liver region; 3) vascular blurring on color Doppler ultrasound; 4) poorly displayed intrahepatic luminal structures. The exclusion criteria for this study were as follows: 1) subjects with a history of drinking, or alcohol consumption ≥140 g per week for men and ≥70 g per week for women; 2) subjects with a history of autoimmune hepatitis, drug-induced hepatic disease, viral hepatitis or other known diseases that may lead to fatty liver; 3) subjects treated with lipid-lowing agents or anti-diabetic medications; 4) subjects who did not receive liver ultrasound; 5) subjects with incomplete clinical information. Finally, 371 patients with newly diagnosed T2DM combined with NAFLD and 360 newly diagnosed T2DM patients without NAFLD were included in this study (**Supplementary Figure 1**).

### Data collection

Basic clinical data and lifestyle information of the study population were collected, including age, sex, height, body weight, and smoking/alcohol consumption habits. Smoking/drinking habits depended on whether the individual currently smoked or drank excessively (140 g/week for men and 70 g/week for women). The levels of blood lipids, blood glucose, liver function and renal function were collected in this study, including alanine aminotransferase (ALT), aspartate aminotransferase (AST), Gamma-glutamyl transferase (GGT), alkaline phosphatase (ALP), serum creatinine (Scr), UA, fasting blood-glucose (FBG), glycosylated hemoglobin (HbA1c), fasting insulin (FINS), TG, TC, free fatty acid (FFA), LDL-C, HDL-C, APOA1 and APOB. The lipid profiles, liver function, renal function and FBG were detected on an automatic biochemical analyzer (AU 5800, Beckman Coulter, USA). HbA1c was assessed by high-performance liquid chromatography (HLC-723G8, TOSOH CORPORATION, Japan). FINS was measured by an automatic electrochemiluminescence immunoassay analyzer (ADVIA centaur XP, Siemens, Germany).

Body mass index (BMI) was calculated as body weight (kg)/height^2^ (m^2^). Homeostasis model assessment-insulin resistance (HOMA-IR) reflects the state of insulin resistance (IR) in the body, and the equation is: HOMA-IR = fasting insulin (μU/dL) × fasting blood glucose (mg/dL)/22.5. TG/HDL-C, TC/HDL-C, FFA/HDL-C, LDL-C/HDL-C, UA/HDL-C, and AOB/A1 ratios were calculated as TG (mmol/L)/HDL-C (mmol/L), TC (mmol/L)/HDL-C (mmol/L), FFA (mmol/L)/HDL-C (mmol/L), LDL-C (mmol/L)/HDL-C (mmol/L), UA (μmol/L)/HDL-C (mmol/L), APOB (mmol/L)/APOA1 (mmol/L) respectively.

### Statistical analysis

Statistical analysis was performed using SPSS 22.0 software. Continuous variables with normal distribution were expressed as mean ± standard deviation (SD), and independent samples T-test was used to compare the non-NAFLD group with the NAFLD group. Continuous variables without a normal distribution were expressed as median (interquartile range), and compared between the non-NAFLD and NAFLD groups using the Kruskal-Wallis test. Categorical variables were shown as proportions, and compared using Chi-squared tests. We divided the TG/HDL-C ratio, TC/HDL-C ratio, FFA/HDL-C ratio, LDL-C/HDL-C ratio, UA/HDL-C and APOB/A1 ratio into four quartiles and converted them into conventional categorical variables, i.e. Q1 < 25%, Q2 25-50%, Q3 50-75% and Q4 ≥ 75%. Chi-square test was used to compare the proportion of NAFLD in patients with newly-onset T2DM in the above categorical variables. Continuous variables that did not conform to a normal distribution were log-transformed, and Pearson correlation analysis was conducted between the six lipid-lipoprotein ratios and each variable. After adjusting for potential confounders, a bivariate logistic regression analysis was performed in newly diagnosed T2DM patients to explore the association between several lipid ratios and NAFLD. Three models were used in this study, model 1 unadjusted; model 2 adjusted for age, sex, current smoking status, and BMI; and model 3 adjusted for age, sex, current smoking status, BMI, ALT, AST, GGT, ALP, Scr, FBG, HbA1c and FINS. The receiver operating characteristic (ROC) curve analysis was used to compare the relative diagnostic ability of the six lipids and lipoprotein ratios for new-onset T2DM combined with NAFLD. The indicator with the largest area under the ROC curve (AUC) was considered the best diagnostic marker.

## Results

### Clinical characteristics of the study subjects

A total of 731 newly diagnosed T2DM subjects were enrolled in the study, including 360 patients without NAFLD (non-NAFLD group), 371 patients with NAFLD (NAFLD group). That is, the overall proportion of NAFLD in patients with newly diagnosed T2DM was 50.8%. In non-NAFLD group, the mean age was 57.21 ± 16.83 years, with 58.9% (212/360) of males and 41.1% (148/360) of females. In NAFLD group, the mean age was 51.45 ± 15.86 years, of which 65.2% (242/371) were males and 34.8% (129/371) were females. Moreover, newly diagnosed T2DM subjects combined with NAFLD smoked more and had a higher BMI than subjects without NAFLD. As expected, patients with NAFLD had higher ALT, AST, GGT, ALP, UA, FBG, FINS, HOMA-IR, TG, TC, FFA, LDL-C and APOB than non-NAFLD group, while HDL-C and APOA1 were lower than non-NAFLD group. There was no significant difference in Scr and HbA1c between the two groups (**Table 1**).

**Table 1:** Clinical characteristics of the study subjects in newly diagnosed T2DM with and without NAFLD.

	Non-NAFLD (N=360)	NAFLD (N=371)	*P*-Values
Age (years)	57.21 ± 16.83	51.45 ± 15.86	<0.001
Sex, male/female (n)	212/148	242/129	0.077
Current smoking (%)	79 (21.9)	113 (30.5)	0.009
BMI (kg/m^2^)	23.65 ± 4.20	27.28 ± 4.82	<0.001
ALT (U/L)	19.30 (13.43-29.4)	30.30 (19.00-55.1)	<0.001
AST (U/L)	18.75 (14.90-24.85)	23.3 (17.4-38.30)	<0.001
GGT (U/L)	25.5 (17.45-38.85)	39.9 (26.60-66.05)	<0.001
ALP (U/L)	91.13 ± 29.98	98.52 ± 38.38	0.008
Scr (μmol/L)	71.45 (60.50-82.98)	74.7 (63.10-74.70)	0.202
UA (μmol/L)	306.06 ± 94.57	343.02 ± 100.63	<0.001
FBG (mmol/L)	9.72 ± 4.19	11.45 ± 5.50	<0.001
HbA1c (%)	10.60 ± 4.20	10.84 ± 2.59	0.280
FINS (μIU/mL)	9.18 (6.07-12.65)	11.72 (8.04-15.79)	<0.001
HOMA-IR	3.43 (2.14-5.62)	5.23 (3.36-7.85)	<0.001
TG (mmol/L)	1.27 (0.92-1.72)	1.85 (1.31-2.85)	<0.001
TC (mmol/L)	4.72 ± 1.15	5.25 ± 1.81	<0.001
FFA (mmol/L)	0.51 ± 0.23	0.58 ± 0.22	<0.001
LDL-C (mmol/L)	3.16 ± 0.91	3.35 ± 1.01	0.006
HDL-C (mmol/L)	1.07 ± 0.24	0.95 ± 0.22	<0.001
APOA1 (mmol/L)	1.16 ± 0.18	1.12 ± 0.18	0.006
APOB (mmol/L)	0.98 ± 0.23	1.09 ± 0.22	<0.001
TG/HDL-C	1.20 (0.84-1.80)	2.05 (1.37-3.23)	<0.001
TC/HDL-C	4.61 ± 1.19	5.57 ± 1.86	<0.001
FFA/HDL-C	0.51 ± 0.26	0.64 ± 0.32	<0.001
LDL-C/HDL-C	3.09 ± 0.92	3.62 ± 1.04	<0.001
UA/HDL-C	303.83 ± 131.20	386.35 ± 159.50	<0.001
APOB/APOA1	0.87 ± 0.22	1.00 ± 0.27	<0.001

Values are expressed as mean ± SD, median (quartile) or number (percentage). NAFLD, non-alcoholic fatty liver disease; T2DM, type 2 diabetes mellitus; BMI, body mass index; ALT, alanine aminotransferase; AST, aspartate aminotransferase; GGT, gamma-glutamyl transferase; ALP, alkaline phosphatase; Scr, serum creatinine; UA, uric acid; FBG, fasting blood-glucose; HbA1c, glycosylated hemoglobin; FINS, fasting insulin; HOMA-IR, homeostasis model assessment-insulin resistance; TG, triglycerides; TC, cholesterol; FFA, free fatty acid; LDL-C, low-density lipoprotein-cholesterol; HDL-C, high-density lipoprotein-cholesterol; APOA1, apolipoprotein A1; APOB, apolipoprotein B; TG/HDL-C, TG to HDL-C ratio; TC/HDL-C, TC to HDL-C ratio; FFA/HDL-C, FFA to HDL-C ratio; UA/HDL-C, UA to HDL-C ratio; LDL-C/HDL-C, LDL-C to HDL-C ratio; APOB/A1, APOB to APOA1 ratio.

The distribution of the ratios of TG/HDL-C, TC/HDL-C, FFA/HDL-C, LDL-C/HDL-C, UA/HDL-C, and APOB/A1 in the non-NAFLD and NAFLD groups, respectively, is shown in **Supplementary Figure 2**. In addition, the ratios of TG/HDL-C, TC/HDL-C, FFA/HDL-C, LDL-C/HDL-C, UA/HDL-C, APOB/A1 were significantly higher in new-onset T2DM patients with NAFLD than in patients without NAFLD (**Table 1**).

### Associations of six lipid and lipoprotein-related indices with NAFLD in newly diagnosed T2DM

**Figure 1:**
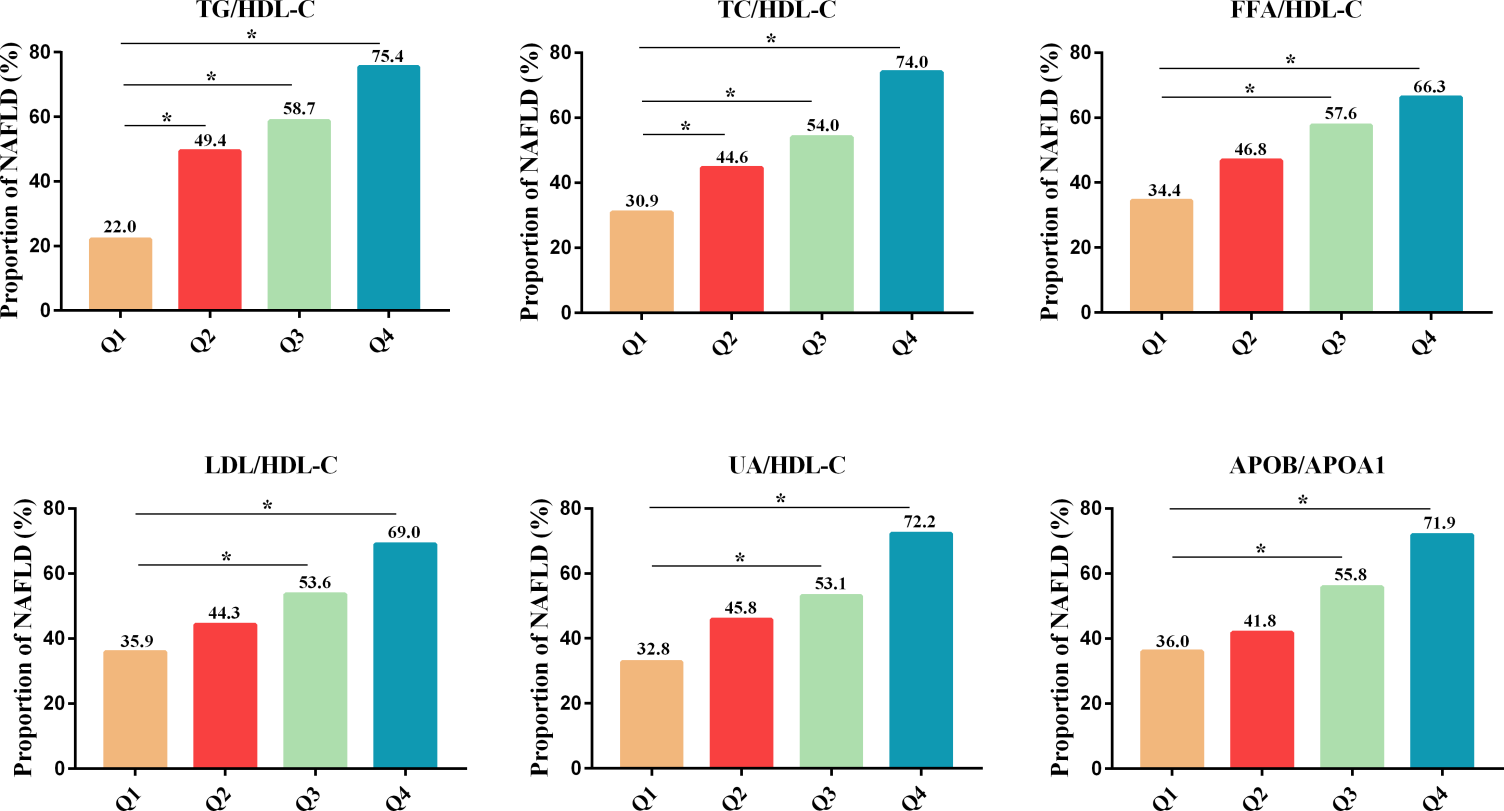
Proportion of NAFLD in patients with newly diagnosed T2DM across the quartiles of multiple lipid ratios (Q1-Q4). *P<0.001 vs. Q1. NAFLD, non-alcoholic fatty liver disease; T2DM, type 2 diabetes mellitus; TG/HDL-C, triglycerides to high-density lipoprotein-cholesterol ratio; TC/HDL-C, cholesterol to HDL-C ratio; FFA/HDL-C, free fatty acid to HDL-C ratio; UA/HDL-C, uric acid to HDL-C ratio; LDL-C/HDL-C, low-density lipoprotein-cholesterol to HDL-C ratio; APOB/A1, apolipoprotein B to apolipoprotein A1 ratio.

The proportion of NAFLD in newly diagnosed T2DM patients increased progressively across the Q1-Q4 range of six lipid-lipoprotein ratios, including TG/HDL-C (22.0, 49.4, 58.7 and 75.4%, respectively), TC/HDL-C (30.9, 44.6, 54.0 and 74.0%, respectively), FFA/HDL-C (34.4, 46.8, 57.6 and 66.3%, respectively), LDL-C/HDL-C (35.9, 44.3, 53.6 and 69.0%, respectively), UA/HDL-C (32.8, 45.8, 53.1 and 72.2%, respectively) and APOB/A1 (36.0, 41.8, 55.8 and 71.9%, respectively) (**Figure 1**). Compared to the lowest quartile (Q1) of the above six lipid-lipoprotein ratios, the proportion of NAFLD was significantly higher in the increasing quartiles (Q2-Q4) of the TG/HDL-C and TC/HDL-C ratios, and in the increasing quartile (Q3-Q4) of the FFA/HDL-C, LDL-C/HDL-C and UA/HDL-C and APOB/A1 ratios (**Figure 1**). This increasing trend suggested that the greater the six lipid ratios in newly diagnosed T2DM patients, the higher the likelihood of NAFLD occurrence in those patients. Logistic regression analysis further demonstrated that the 6 lipid ratios in model 1 were positively correlated with NAFLD in new-onset T2DM patients without adjusting for other factors (**Table 2**). Pearson correlation analysis was shown in **Supplementary Table 1**, indicating that the 6 lipid ratios were strongly correlated with age, sex, BMI, hepatic function markers, renal function indicators, blood glucose indicators and blood lipid indicators, respectively. Therefore, we next corrected for these factors in models 2 and 3 of the logistic regression analysis (**Table 2**). After adjusting for age, sex, current smoking status and BMI in model 2, the 6 lipid ratios remained significantly and positively associated with NAFLD in newly diagnosed T2DM patients (**Table 2**). Even after adjusting for age, sex, current smoking status, BMI, ALT, AST, GGT, ALP, Scr, FBG, HbA1c and FINS in model 3, 5 lipid ratios (TG/HDL-C, TC/HDL-C, LDL-C/HDL-C, UA/HDL-C, and APOB/A1) remained positively associated with the risk of NAFLD in patients with newly diagnosed T2DM (**Table 2**). It should be noted that in model 1-3, APOB/A1 ratio had the strongest correlation with NAFLD in patients with newly diagnosed T2DM [model1 odds ratio (OR)= 10.72, *P*<0.001; model2 OR=4.81, *P*=0.001 and model3 OR= 6.25; *P*=0.006, respectively] (**Table 2**).

**Table 2:** The association between the lipid ratios and the risk of NAFLD in patients with newly diagnosed T2DM.

	Model1		Model2		Model3	
	OR (95% CI)	*P*-Values	OR (95% CI)	*P*-Values	OR (95% CI)	*P*-Values
TG/HDL-C	1.62 (1.42-1.86)	<0.001	1.49 (1.25-1.77)	<0.001	1.45 (1.14-1.85)	0.002
TC/HDL-C	1.67 (1.46-1.90)	<0.001	1.41 (1.19-1.67)	<0.001	1.43 (1.11-1.83)	0.005
FFA/HDL-C	6.25 (3.40-11.49)	<0.001	2.35 (1.16-4.77)	0.018	1.41 (0.51-3.88)	0.508
LDL-C/HDL-C	1.74 (1.48-2.04)	<0.001	1.40 (1.13-1.73)	0.002	1.51 (1.09-2.08)	0.012
UA/HDL-C	1.00 (1.00-1.01)	<0.001	1.00 (1.00-1.00)	0.006	1.00 (1.00-1.01)	0.016
APOB/APOA1	10.72 (5.29-21.72)	<0.001	4.81 (1.93-12.01)	0.001	6.25 (1.71-22.79)	0.006

Model 1 is unadjusted.

Model 2 is adjusted for age, sex, current smoking, BMI.

Model 3 is adjusted for age, sex, current smoking, BMI, ALT, AST, GGT, ALP, Scr, FBG, HbA1c, FINS NAFLD, non-alcoholic fatty liver disease; T2DM, type 2 diabetes mellitus; BMI, body mass index; ALT, alanine aminotransferase; AST, aspartate aminotransferase; GGT, gamma-glutamyl transferase; ALP, alkaline phosphatase; Scr, serum creatinine; FBG, fasting blood-glucose; HbA1c, glycosylated hemoglobin; FINS, fasting insulin; TG/HDL-C, triglycerides to high-density lipoprotein-cholesterol ratio; TC/HDL-C, cholesterol to HDL-C ratio; FFA/HDL-C, free fatty acid to HDL-C ratio; UA/HDL-C, uric acid to HDL-C ratio; LDL-C/HDL-C, low-density lipoprotein-cholesterol to HDL-C ratio; APOB/A1, apolipoprotein B to apolipoprotein A1 ratio.

### Diagnostic value of the six lipid-lipoprotein ratios for NAFLD in newly diagnosed T2DM patients

**Figure 2:**
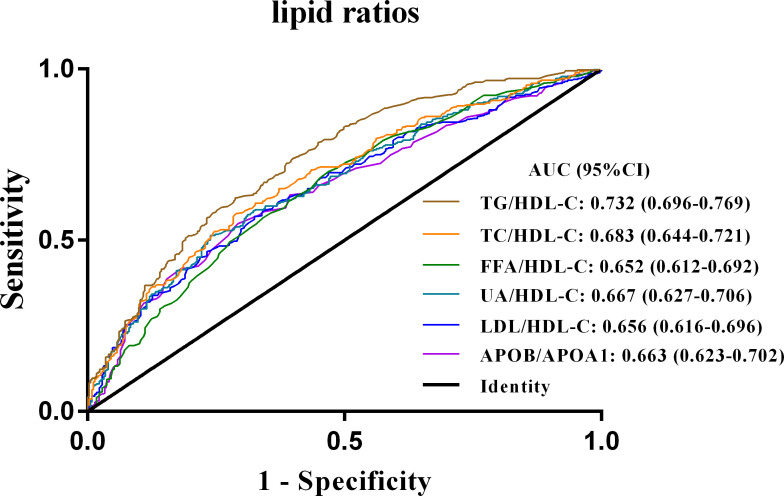
ROC curves of the six lipid ratios in patients with newly diagnosed T2DM combined with NAFLD. TG/HDL-C, triglycerides to high-density lipoprotein-cholesterol ratio; TC/HDL-C, cholesterol to HDL-C ratio; FFA/HDL-C, free fatty acid to HDL-C ratio; UA/HDL-C, uric acid to HDL-C ratio; LDL-C/HDL-C, low-density lipoprotein-cholesterol to HDL-C ratio; APOB/A1, apolipoprotein B to apolipoprotein A1 ratio; ROC curves, receiver operator characteristic curves; NAFLD, non-alcoholic fatty liver disease; T2DM, type 2 diabetes mellitus.

ROC curves were then constructed to compare the ability of the six lipid-lipoprotein ratios and their associated lipid metrics to discriminate NAFLD in newly diagnosed T2DM patients (**Supplementary Figure 3**). The area under the curve (AUC) of all lipid ratios was higher than that of individual lipid indicators, indicating that lipid ratios were superior to individual lipid values in the diagnosis of NAFLD in newly diagnosed T2DM patients (**Supplementary Figure 3**). Furthermore, the results of the ROC curve analysis corresponding to the six lipid ratios were shown in **Figure 2** and **Table 3**, with the highest AUC for the TG/HDL-C ratio (AUC 0.732; 95% CI 0.696-0.769). Moreover, the sensitivity of the TG/HDL ratio (73.8%) was also the highest among all six indicators, with a specificity of 60.1% and a cut-off point of 1.405 (**Table 3**).

**Table 3:** ROC curves of six lipid ratios for the diagnosis of NAFLD in patients with new-onset T2DM.

	AUC	95% CI	*P*-Values	Youden index	Cut-off point	Sensitivity	Specificity
TG/HDL-C	0.732	0.696-0.769	<0.001	0.339	1.405	0.738	0.601
TC/HDL-C	0.683	0.644-0.721	<0.001	0.289	5.175	0.575	0.714
FFA/HDL-C	0.652	0.612-0.692	<0.001	0.232	0.475	0.701	0.532
LDL-C/HDL-C	0.656	0.616-0.696	<0.001	0.245	3.485	0.569	0.675
UA/HDL-C	0.667	0.627-0.706	<0.001	0.275	360.2	0.514	0.761
APOB/APOA1	0.663	0.623-0.702	<0.001	0.280	0.945	0.573	0.767

NAFLD, non-alcoholic fatty liver disease; T2DM, type 2 diabetes mellitus; TG/HDL-C, triglycerides to high-density lipoprotein-cholesterol ratio; TC/HDL-C, cholesterol to HDL-C ratio; FFA/HDL-C, free fatty acid to HDL-C ratio; UA/HDL-C, uric acid to HDL-C (UA/HDL-C) ratio; LDL-C/HDL-C, low-density lipoprotein-cholesterol to HDL-C; APOB/A1, apolipoprotein B to apolipoprotein A1.

**Figure 3:**
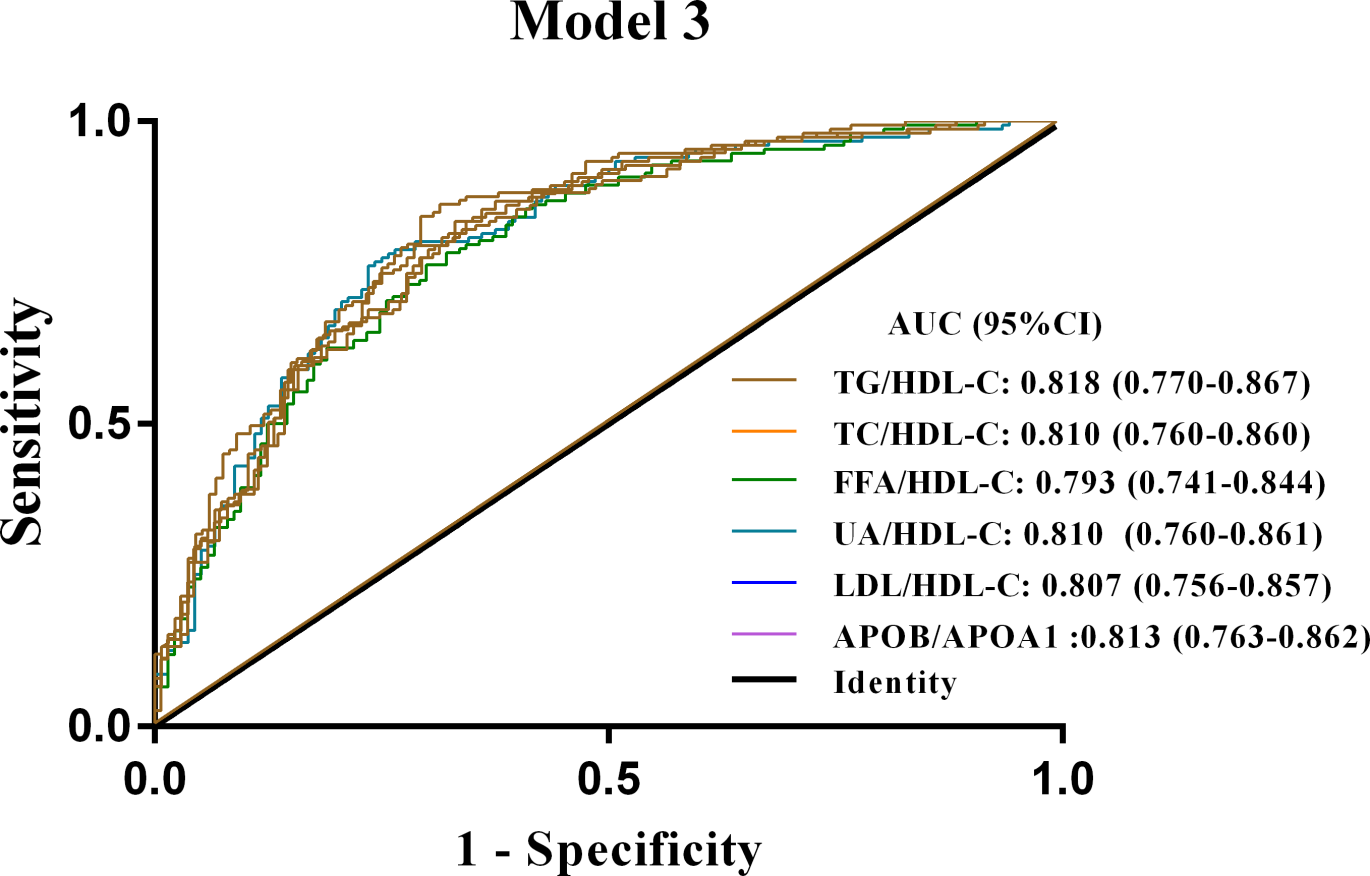
ROC curves for Model 3 of the six lipid ratios in patients with newly diagnosed T2DM combined with NAFLD. Model 3 is adjusted for age, sex, current smoking, BMI, ALT, AST, GGT, ALP, Scr, FBG, HbA1c and FINS. NAFLD, non-alcoholic fatty liver disease; T2DM, type 2 diabetes mellitus; ROC curves, receiver operator characteristic curves; TG/HDL-C, triglycerides to high-density lipoprotein-cholesterol ratio; TC/HDL-C, cholesterol to HDL-C ratio; FFA/HDL-C, free fatty acid to HDL-C ratio; UA/HDL-C, uric acid to HDL-C ratio; LDL-C/HDL-C, low-density lipoprotein-cholesterol to HDL-C ratio; APOB/A1, apolipoprotein B to apolipoprotein A1 ratio.

In addition, ROC curve analysis showed that all six metrics in model 3 had the highest ability to discriminate NAFLD in newly diagnosed T2DM patients among the three models (**Supplementary Figure 4**). Furthermore, after correction for age, gender, current smoking status, BMI, ALT, AST, GGT, ALP, Scr, FBG, HbA1c and FINS, the AUC of the TG/HDL-C ratio in model 3 remained the largest (AUC of 0.818; *P* < 0.001) (**Figure 3**). These results suggested that the TG/HDL ratio was the most promising diagnostic indicator of NAFLD in patients with new-onset T2DM after adjusting for patient age, sex, BMI, current smoking, or biochemical values.

## Discussion

Early detection of NAFLD in patients with newly diagnosed T2DM is of great significance for the implementation of early intervention strategies. However, the invasiveness of liver biopsy or the limitations of the expertise of ultrasound technicians and ultrasound instrumentation have made it difficult to use the above screening methods widely in the general population. Recent studies have found that lipid and lipoprotein disorders promote the development and progression of NAFLD (21, 22). Accumulating clinical evidence have indicated that dyslipidemia and lipoprotein disorders are associated with NAFLD in different populations (8–13, 16, 17), suggesting the possibility of lipid indices or lipid-lipoprotein ratios as diagnostic markers for NAFLD. In this study, we explored the efficacy of six lipid-lipoprotein ratio parameters (TG/HDL-C, TC/HDL-C, FFA/HDL-C, UA/HDL-C, LDL-C/HDL-C, APOB/A1) and their individual lipid indexes for the diagnosis of NAFLD in patients with newly diagnosed T2DM. Our study showed that all lipid-lipoprotein ratios were superior to individual lipid indices in the diagnosis of NAFLD in patients with newly-onset T2DM.

Previous studies on the correlation between lipid-lipoprotein ratios and NAFLD have been reported (9, 11, 16, 17, 23). Ren et al. (11) concluded that the TC/HDL-C ratio had a significant predictive value for NAFLD, and ROC analysis showed that the AUC (0.645) was greater than other serum lipids. In addition, Zhu et al. (9) suggested that the predictive value of UA/HDL-C was significantly higher than LDL-C/HDL-C, non-HDL-C/HDL-C and ALT/AST in a non-obese population, even when UA and LDL-C levels were within the normal range. In a 5-year longitudinal cohort study of 9767 non-obese subjects with normal lipids, Cox proportional hazard regression model confirmed that high LDL-C/HDL-C ratios significantly increased the risk of NAFLD in non-obese Chinese subjects with normal lipids (16). In addition, the APOB/A1 ratio was also associated with the prevalence of NAFLD in non-diabetic subjects (23), normal weight and overweight subjects (17). Although the correlation between lipid-lipoprotein ratio and NAFLD has been reported in non-obese, non-diabetic populations, the correlation between lipid- lipoprotein ratio and NAFLD in newly diagnosed T2DM patients has not been studied.

There is growing evidence revealed a strong association between TG/HDL-C and multiple metabolic disorders, including IR, diabetes, and cardiometabolic risk (13, 18, 24). For example, in a study investigating the relationship between lipid ratios and abnormal glucose tolerance, Guo et al. (18) found that serum TC, TG, TC/HDL-C, TG/HDL-C, and non-HDL-C were all strongly associated with prediabetes and T2DM. The AUC values of both TG and TG/HDL-C exceeded 0.70 in the diagnosis of prediabetes and T2DM. In addition, some studies have found a correlation between TG/HDL-C and NAFLD. For example, a retrospective study demonstrated that TG/HDL-C was independently associated with NAFLD in subjects undergoing health screening and could be used as a surrogate marker for NAFLD (12). In another retrospective cohort study of a Chinese non-obese population without dyslipidemia, there was an independent correlation between TG/HDL-C and NAFLD (10). Although previous studies have identified correlations between TG/HDL-C and NAFLD in physical examination subjects and non-obese populations, no study has so far focused on the diagnostic value of TG/HDL-C for NAFLD in a newly diagnosed T2DM population. Our study suggests for the first time that TG/HDL-C may be a promising biomarker for early identification of NAFLD in newly diagnosed T2DM patients. We found that in patients with newly diagnosed T2DM, TG/HDL-C had an AUC of 0.732, a sensitivity of 73.8% and a specificity of 60.1% for identifying NAFLD, which was significantly higher than other five lipid- lipoprotein ratios.

Our study found that TG/HDL-C might have the potential to be used as a diagnostic indicator of NAFLD in newly diagnosed T2DM. The mechanism of the intrinsic association of TG/HDL-C with T2DM combined with NAFLD may be related to IR. Previous studies have revealed the strong correlation between TG/HDL-C and IR (25–28). And the onset of NAFLD and T2DM are also closely associated with IR (28–32). Excess fatty acids are produced due to increased lipolysis and enhanced fatty acid synthesis. These fatty acids enter the blood circulation and accumulate in peripheral tissues, such as the liver and adipose tissue, ultimately leading to IR (31). In addition, IR also enhances new lipogenesis in the liver and lipolysis in adipose tissue, thereby increasing the amount of fatty acids flowing to the liver (32). Lipids accumulate in the liver in the form of FFA-derived TG, which together with high levels of free cholesterol and lipid metabolites (e.g., unsaturated fatty acids, lipid peroxidation products, etc.), increase lipotoxicity (32, 33). Also, β-cell failure due to excess free fatty acids and lipid metabolites, as well as IR, are major pathogenic mechanisms of T2DM (33). The molecular mechanisms underlying the association between TG/HDL-C and the risk of NAFLD in newly diagnosed T2DM still deserve further exploration.

There are some limitations of our study. Firstly, it is uncertain whether the TG/HDL-C ratio remains a diagnostic indicator for NAFLD in patients with longer duration of T2DM. Follow-up studies of these patients will be able to clarify this issue. Secondly, all patients with T2DM recruited in this study were newly diagnosed and had not received oral lipid-lowering or hypoglycemic medications. The strict inclusion criteria resulted in a small sample size for inclusion. Thirdly, some newly diagnosed T2DM patients were not included in this study due to the lack of liver ultrasound imaging, which may lead to some degree of data bias.

## Conclusion

In summary, this is the first study to assess the diagnostic value of multiple simple lipid parameter ratios for NAFLD in newly diagnosed T2DM patients. Our results found that the proportion of NAFLD in newly diagnosed T2DM patients elevated progressively with increasing ratios of six lipid parameters. Our study suggest that the TG/HDL-C ratio has the best diagnostic value for NAFLD in the newly diagnosed T2DM population, and may has the potential to be used as a screening marker for NAFLD in the newly diagnosed T2DM population in clinical practice and in large-scale screening.

## Data availability statement

The original contributions presented in the study are included in the article/**Supplementary Material**. Further inquiries can be directed to the corresponding author.

## Ethics statement

The studies involving human participants were reviewed and approved by the Ethics Committee of Tongji Hospital, Tongji University School of Medicine (K-2021-010). Written informed consent for participation was not required for this study in accordance with the national legislation and the institutional requirements.

## Author contributions

RL and DK analyzed the patient data and drafted the manuscript. ZY and GZ contributed to data interpretation. KH, WX, PF coordinated the research. LZ and YZ contributed to data interpretation and critical revision of the manuscript. KZ and YX designed the study, revised and prepared the final version of the manuscript. All authors read and approved the final manuscript. All authors contributed to the article and approved the submitted version.
