# Supplementary material for: Correlation of multiple lipid and lipoprotein ratios with nonalcoholic fatty liver disease in patients with newly diagnosed type 2 diabetic mellitus: A retrospective study
Source: Front Endocrinol (Lausanne). 2023 Feb 17;14:1127134. doi: 10.3389/fendo.2023.1127134 (PMC9982122; doi:10.3389/fendo.2023.1127134)
Supplement: Supplementary file 1 [file DataSheet_1.docx]

**Supplementary Table 1** Pearson correlations between lipid ratios and clinical variables in all patients

|  | TG/HDL-C | | TC/HDL-C | | FFA/HDL-C | | LDL-C/HDL-C | | UA/HDL-C | | APOB/APOA1 | |
| --- | --- | --- | --- | --- | --- | --- | --- | --- | --- | --- | --- | --- |
|  | *r* | *p* | *r* | *p* | *r* | *p* | *r* | *p* | *r* | *p* | *r* | *p* |
| Age | -0.235 | ＜0.001 | -0.291 | ＜0.001 | -0.213 | ＜0.001 | -0.258 | ＜0.001 | -0.281 | ＜0.001 | -0.186 | ＜0.001 |
| Female | -0.151 | ＜0.001 | -0.176 | ＜0.001 | -0.123 | 0.001 | -0.157 | ＜0.001 | -0.295 | ＜0.001 | -0.101 | 0.007 |
| BMI | 0.300 | ＜0.001 | 0.389 | ＜0.001 | 0.291 | ＜0.001 | 0.332 | ＜0.001 | 0.423 | ＜0.001 | 0.315 | ＜0.001 |
| ALT | 0.300 | ＜0.001 | 0.207 | ＜0.001 | 0.154 | ＜0.001 | 0.185 | ＜0.001 | 0.286 | ＜0.001 | 0.175 | ＜0.001 |
| AST | 0.229 | ＜0.001 | 0.192 | ＜0.001 | 0.148 | ＜0.001 | 0.117 | ＜0.001 | 0.223 | ＜0.001 | 0.151 | ＜0.001 |
| GGT | 0.325 | ＜0.001 | 0.230 | ＜0.001 | 0.073 | 0.073 | 0.251 | ＜0.001 | 0.234 | ＜0.001 | 0.187 | ＜0.001 |
| ALP | 0.116 | ＜0.001 | 0.118 | ＜0.001 | 0.039 | 0.341 | 0.157 | ＜0.001 | 0.089 | ＜0.001 | 0.172 | ＜0.001 |
| Scr | 0.160 | ＜0.001 | 0.071 | 0.057 | 0.117 | 0.002 | 0.075 | 0.045 | 0.376 | ＜0.001 | 0.112 | 0.002 |
| UA | 0.405 | ＜0.001 | 0.327 | ＜0.001 | 0.231 | ＜0.001 | 0.251 | ＜0.001 | 0.830 | ＜0.001 | 0.269 | ＜0.001 |
| FBG | 0.233 | ＜0.001 | 0.202 | ＜0.001 | 0.230 | ＜0.001 | 0.230 | ＜0.001 | 0.124 | 0.002 | 0.197 | ＜0.001 |
| HbA1c | 0.064 | 0.123 | 0.148 | ＜0.001 | 0.081 | 0.049 | 0.174 | ＜0.001 | -0.003 | 0.943 | 0.158 | ＜0.001 |
| FINS | 0.175 | ＜0.001 | 0.093 | 0.023 | 0.020 | 0.630 | 0.078 | 0.059 | 0.185 | ＜0.001 | 0.077 | 0.059 |
| HOMA-IR | 0.304 | ＜0.001 | 0.229 | ＜0.001 | 0.161 | ＜0.001 | 0.229 | ＜0.001 | 0.229 | ＜0.001 | 0.187 | ＜0.001 |
| TG | 0.926 | ＜0.001 | 0.696 | ＜0.001 | 0.347 | ＜0.001 | 0.227 | ＜0.001 | 0.377 | ＜0.001 | 0.149 | ＜0.001 |
| TC | 0.388 | ＜0.001 | 0.641 | ＜0.001 | -0.046 | 0.215 | 0.436 | ＜0.001 | -0.045 | 0.232 | 0.354 | ＜0.001 |
| FFA | 0.237 | ＜0.001 | 0.181 | ＜0.001 | 0.822 | ＜0.001 | 0.094 | 0.013 | 0.145 | ＜0.001 | 0.110 | 0.003 |
| LDL-C | -0.028 | 0.463 | 0.445 | ＜0.001 | -0.100 | 0.008 | 0.698 | ＜0.001 | -0.100 | 0.008 | 0.495 | ＜0.001 |
| HDL-C | -0.345 | ＜0.001 | -0.441 | ＜0.001 | -0.476 | ＜0.001 | -0.457 | ＜0.001 | -0.652 | ＜0.001 | -0.266 | ＜0.001 |
| APOA1 | -0.185 | ＜0.001 | -0.338 | ＜0.001 | -0.278 | ＜0.001 | -0.350 | ＜0.001 | -0.393 | ＜0.001 | -0.504 | ＜0.001 |
| APOB | 0.019 | 0.609 | 0.371 | ＜0.001 | 0.021 | 0.567 | 0.538 | ＜0.001 | 0.112 | 0.003 | 0.797 | ＜0.001 |

Abbreviations: BMI, body mass index; ALT, alanine aminotransferase; AST, aspartate aminotransferase; GGT, gamma-glutamyl transferase; ALP, alkaline phosphatase; Scr, serum creatinine; UA, uric acid; FBG, fasting blood-glucose; HbA1c, glycosylated hemoglobin; FINS, fasting insulin; HOMA-IR, homeostasis model assessment-insulin resistance; TG, triglycerides; TC, cholesterol; FFA, free fatty acid; LDL-C, low-density lipoprotein-cholesterol; HDL-C, high-density lipoprotein-cholesterol; APOA1, apolipoprotein A1; APOB, apolipoprotein B; TG/HDL-C, TG to HDL-C ratio; TC/HDL-C, TC to HDL-C ratio; FFA/HDL-C, FFA to HDL-C ratio; UA/HDL-C, UA to HDL-C ratio; LDL-C/HDL-C, LDL-C to HDL-C ratio; APOB/A1, APOB to APOA1 ratio.

**
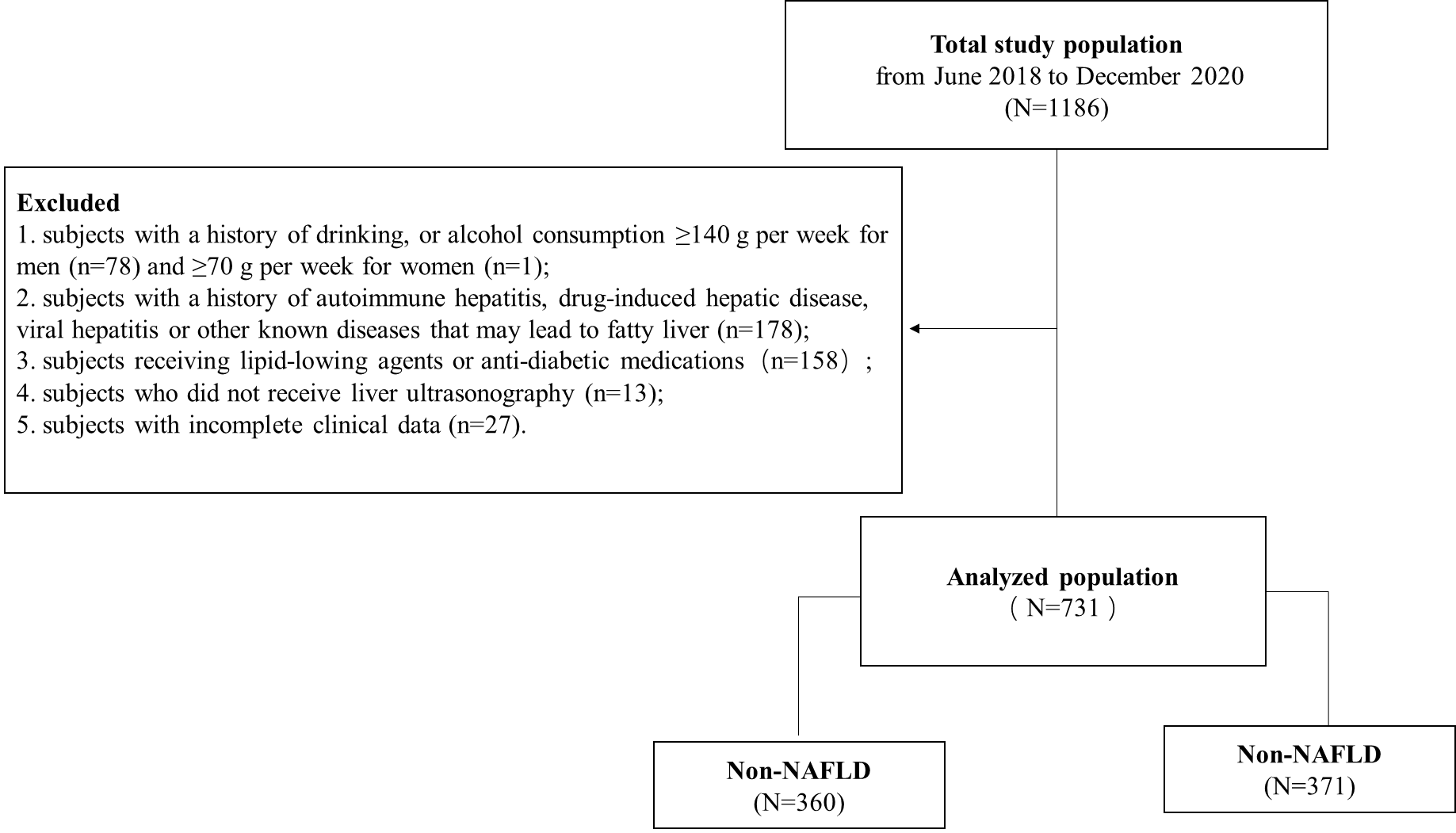
**

**Supplementary Figure 1.** Flow chart of the participants’ inclusion and exclusion in this study.


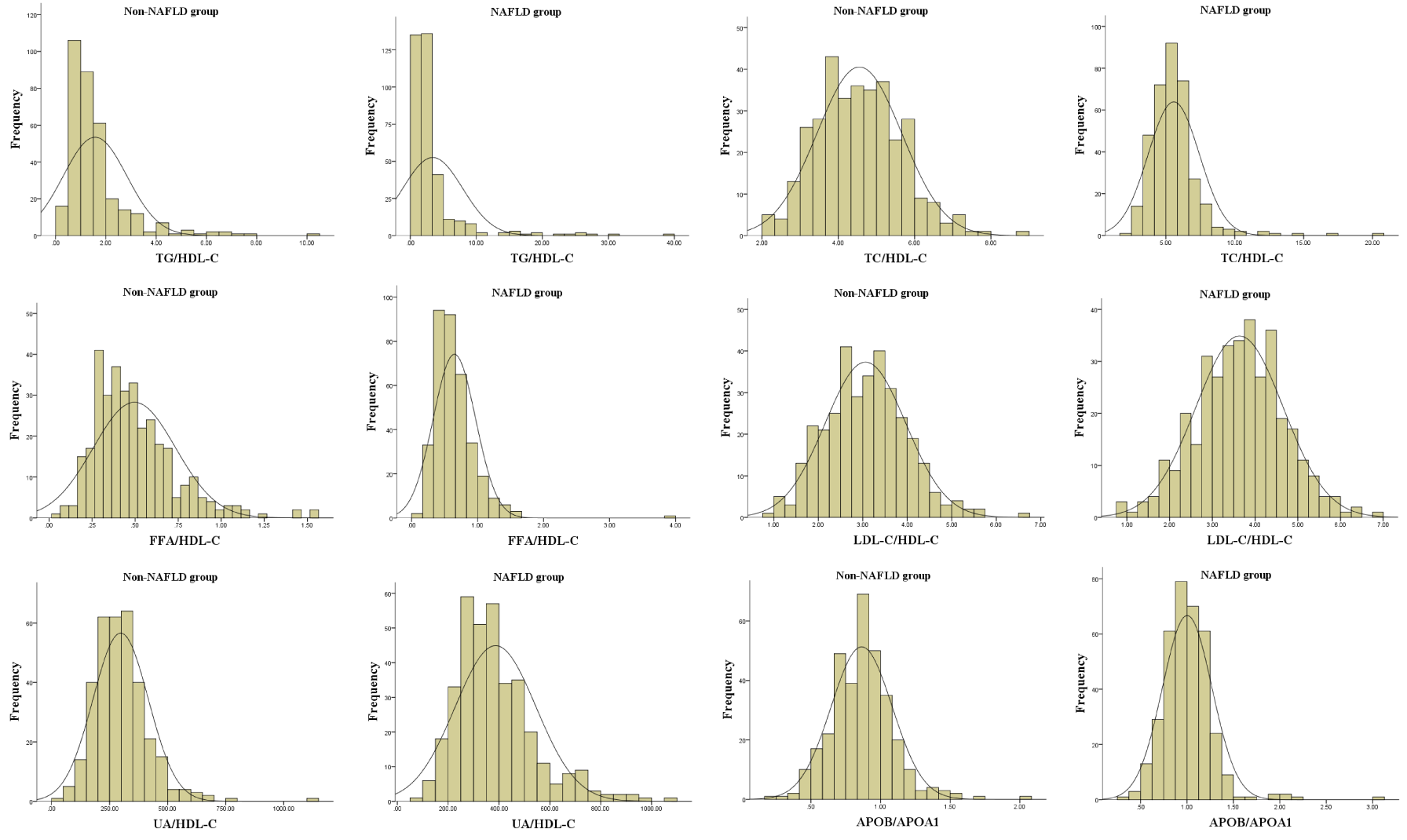


**Supplementary Figure 2.** The distribution of the six lipid ratios in the non-NAFLD and NAFLD groups, respectively.

Abbreviations: TG/HDL-C, triglycerides to high-density lipoprotein-cholesterol ratio; TC/HDL-C, cholesterol to HDL-C ratio; FFA/HDL-C, free fatty acid to HDL-C ratio; LDL-C/HDL-C, low-density lipoprotein-cholesterol to HDL-C ratio; UA/HDL-C, uric acid to HDL-C ratio；APOB/A1, apolipoprotein B to apolipoprotein A1 ratio; NAFLD, non-alcoholic fatty liver disease.


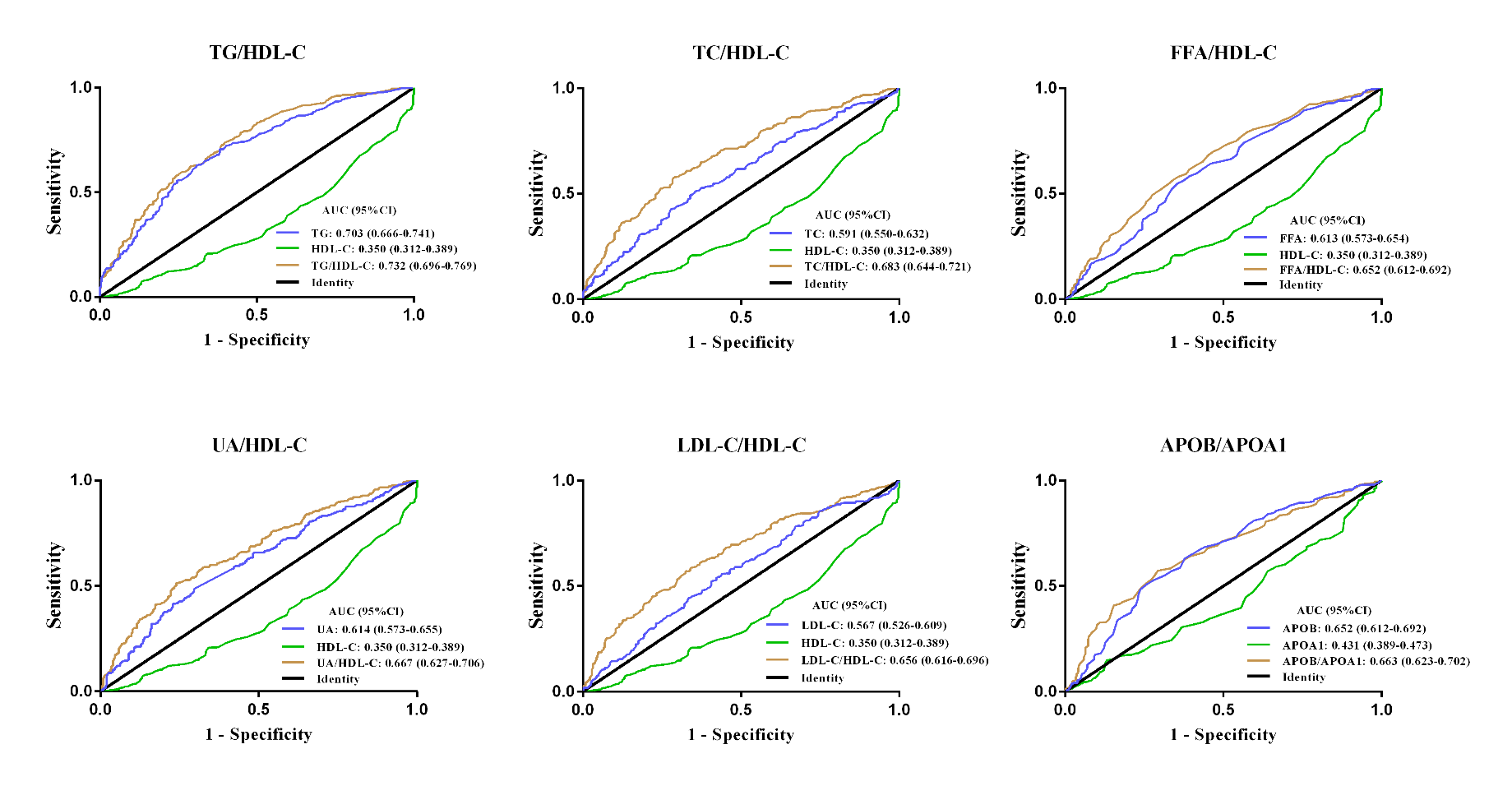


**Supplementary Figure 3.** ROC curves for the six lipid ratios and individual lipids in newly diagnosis T2DM with NAFLD.

Abbreviations: TG, triglycerides; TC, cholesterol; FFA, free fatty acid; UA, uric acid; LDL-C, low-density lipoprotein-cholesterol; HDL-C, high-density lipoprotein-cholesterol; APOA1, apolipoprotein A1; APOB, apolipoprotein B; TG/HDL-C, TG to HDL-C ratio; TC/HDL-C, TC to HDL-C ratio; FFA/HDL-C, FFA to HDL-C ratio; UA/HDL-C, UA to HDL-C ratio; LDL-C/HDL-C, LDL-C to HDL-C ratio; APOB/A1, APOB to APOA1 ratio; ROC curves, receiver operator characteristic curves; NAFLD, non-alcoholic fatty liver disease; T2DM, type 2 diabetes mellitus.


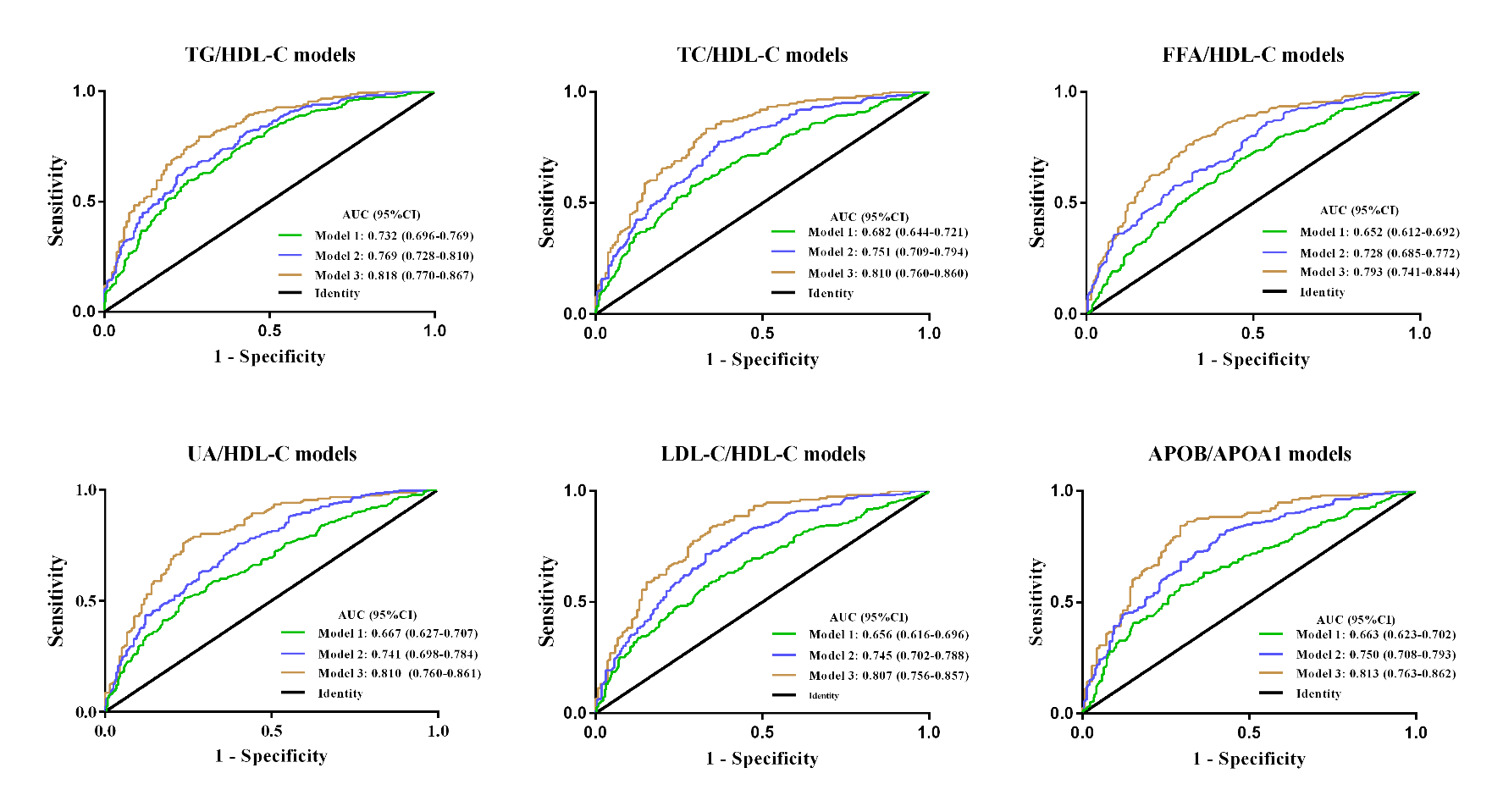


**Supplementary Figure 4.** ROC curves for Models 1–3 of the six lipid ratios in newly diagnosis T2DM with NAFLD.

Abbreviations: TG/HDL-C, triglycerides to high-density lipoprotein-cholesterol ratio; TC/HDL-C, cholesterol to HDL-C ratio; FFA/HDL-C, free fatty acid to HDL-C ratio; LDL-C/HDL-C, low-density lipoprotein-cholesterol to HDL-C ratio; UA/HDL-C, uric acid to HDL-C ratio；APOB/A1, apolipoprotein B to apolipoprotein A1 ratio; ROC curves, receiver operator characteristic curves; NAFLD, non-alcoholic fatty liver disease; T2DM, type 2 diabetes mellitus.
